# Comparison of Measurements of 12 Analytes in Equine Blood Samples Using the In-Practice Falcor 350 and the Reference KoneLab 30i Analysers

**DOI:** 10.5402/2012/475419

**Published:** 2012-09-27

**Authors:** K. Papasouliotis, K. V. Tennant, S. Dodkin, J. Mason

**Affiliations:** ^1^Diagnostic Laboratories, Langford Veterinary Services, University of Bristol, Langford, Bristol BS40 5DU, UK; ^2^School of Veterinary Science, University of Bristol, Langford, Bristol BS40 5DU, UK; ^3^Equine Centre, Langford Veterinary Services, University of Bristol, Langford, Bristol BS40 5DU, UK

## Abstract

Falcor 350 is a wet-reagent biochemistry analyser that is available for in-house use. The aim of this study was to compare the results produced by this analyser with those obtained by the KoneLab 30i that served as the reference instrument. Blood samples from 60 clinical cases were analysed for urea, creatinine, total proteins, albumin, creatine kinase, aspartate aminotransferase, alkaline phosphatase, total bilirubin, total calcium, phosphate, sodium, and potassium using both instruments. Good to excellent correlations (*r*_*s*_ value) value) were identified for creatinine (0.88), total proteins (0.92), albumin (0.93), creatine kinase (0.98), aspartate aminotransferase (0.98), alkaline phosphatase (0.94), total bilirubin (0.98), phosphate (0.95), and potassium (0.97). The correlations for total calcium (0.71), sodium (0.68), and urea (0.64) were fair. For albumin, aspartate aminotransferase, creatine kinase, phosphate, potassium, total bilirubin, creatinine, and total proteins, the two instruments produce values that are closely related to each other and are sufficiently similar to allow them to be used interchangeably without the need for additional correction factor computations. Because of differences in the methodologies, the Falcor results for alkaline phosphatase, total calcium, and sodium cannot be used interchangeably and should be interpreted using reference intervals established from the Falcor analyser.

## 1. Introduction

In veterinary practice, the greatest advantage of performing laboratory analyses in-house is that the results are available immediately. In equine practice, the most widely used in-house biochemistry analysers are instruments which use slides or strips with dry reagents (dry-reagent analysers), and over the years, studies have been published comparing the results produced by these instruments with those obtained by reference analysers [1–5]. Recently, wet-reagent analysers are becoming popular for use in veterinary practices [6]. These instruments operate on the same principles of analysis as the dry-chemistry analysers but instead of using dry reagents, they use liquid reagents which are significantly cheaper. Wet-chemistry analysers require higher capital investment; their reagents once opened are stable for a shorter time than those of the dry-chemistry systems, and in order to be cost-effective, a large number of chemistries have to be run every day. Although wet-chemistry units are more technically demanding to use, the manufacturers have continued to make the instruments simpler in order to remain competitive in the market with the dry-chemistry units. A new wet-reagent biochemistry analyser, the Falcor 350 (A. Menarini Diagnostics), has become available for in-house use. At present, there are no published studies in the equine literature comparing the Falcor with a reference analyser. The aim of this study was to compare the results produced by the analyser with those obtained by a wet-reagent analyser that is used routinely in the authors' laboratory and this served as the reference instrument.

## 2. Materials and Methods

### 2.1. Samples

Blood samples from 60 clinical cases, submitted to the Diagnostic Laboratory at the University of Bristol over a two-month period, were included in the study. We utilised samples from clinical cases in order to generate a wide range of values which can more accurately provide information about the degree of correlation and agreement between the two instruments. The horses showed a variety of clinical signs and had been presented to the Equine Centre for further investigation. Venous blood samples were collected into plain tubes and serum samples were analysed for 12 analytes including urea (mmol/L), creatinine (*μ*mol/L), total proteins (TP, g/L), albumin (g/L), creatine kinase (CK, mmol/L), aspartate aminotransferase (AST, iu/L), alkaline phosphatase (ALP, iu/L), total bilirubin (TB, *μ*mol/L), total calcium (tCa, mmol/L), phosphate (mmol/L), sodium (Na^+^, mmol/L), and potassium (K^+^, mmol/L) using both the reference (KoneLab 30i; Thermo Clinical Labsystems) and Falcor instruments. The samples were run once on each analyser following standard methodologies (Table 1) by the same experienced clinical biochemist (SD) according to the manufacturers' instructions (KoneLab 30i Reference Manual and Falcor User's Guide) within one hour of blood collection. A “multisera” precision normal control sample (Randox) was run daily on the Falcor, while control samples (Biostat Diagnostic Systems) with low, normal, and high values were included in every run of samples in the KoneLab analyser. All reagents used for each instrument during the study were from the same manufacturer's batch. The KoneLab was operated and maintained by clinical biochemists and biochemistry technicians and the accuracy of its methodologies was assessed by continuous bimonthly participation in an external quality assurance programme (RIQAS; Randox).

### 2.2. Statistical Analysis

Bland-Altman plots and Deming regression were performed using the Prism 4 for Macintosh programme (GraphPad software Inc., San Diego, CA, USA). All other data analysis was performed using the SPSS 12.0 for Windows statistical programme (SPSS Inc., Chicago, IL, USA). As the data was not normally distributed, the correlation between methods was determined using Spearman's correlation coefficient. Based on an objective classification system used in previous studies [6–8], correlation was characterised as excellent (*r* ≥ 0.93), good (*r* = 0.80 to 0.92), fair (*r* = 0.59 to 0.79), or poor (*r* < 0.59). Values (*r*_*s*_) greater than 0.80 were considered to indicate acceptable correlation for a new laboratory test compared with a reference method [9, 10]. The degree of agreement between the two analysers was evaluated by examination of the Bland-Altman difference plots. Agreement was considered good when there was no real bias (mean of the differences, Falcor—KoneLab) or the bias was small, the 95% confidence intervals for the bias were narrow and no outliers were present (no values fell outside the limits of agreement (mean of the differences ± 2SD)). No real bias was indicated when the 95% confidence intervals (CI) for the bias included zero [11–13].

## 3. Results

**Figure 1:**
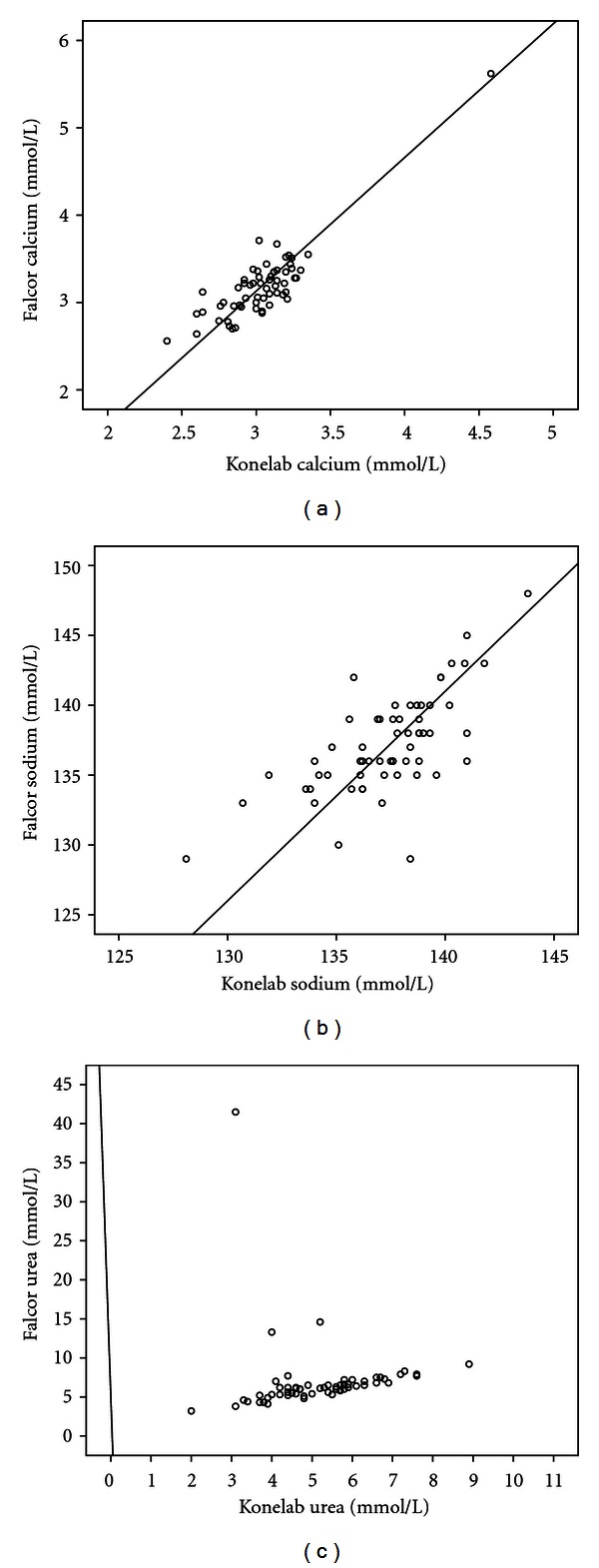
Deming regression plots of KoneLab versus Falcor analysers for tCa, Na^+^, and urea. The solid line represents the best fitted line.

Correlations between the Falcor and KoneLab results were excellent for 7 analytes (albumin, ALP, AST, CK, phosphate, K^+^, and TB), good for 2 analytes (creatinine, TP), and fair for tCa, Na^+^, and urea measurements (Table 2, Figures 1(a)–1(c)).

Examination of the Bland-Altman difference plots revealed that results from the two instruments were in good agreement (not real or small bias, narrow 95% CI for the bias, no outliers) for 8 analytes (albumin, AST, CK, phosphate, K^+^, TB, creatinine, and TP; Table 3). For ALP, the agreement was poor (real large bias with wide 95% CI and 4 outliers) as the Falcor generated higher ALP results than the KoneLab (Table 3).

Regression plots for results with acceptable correlation (*r*_*s*_ > 0.80) and good agreement with unremarkable Bland-Altman plots are not shown.

## 4. Discussion

Examination of the regression and difference plots revealed excellent correlation and good agreement for 8 of the 12 analytes (albumin, AST, CK, phosphate, K^+^, TB, creatinine, and TP).

For tCa, there was a statistically unacceptable correlation between the two instruments. Examination of the data revealed that in 45 of the 60 cases (75%), the Falcor generated tCa values higher than those of the KoneLab. This finding may reflect the different methodologies employed by the two instruments (Cresolphthalein complexone versus Arsenazo III) and is in agreement with the results reported in an identical study in cats and dogs where in 49 of 60 canine (81%) and 39 of 60 feline (65%) cases the Falcor generated tCa values higher than those of the KoneLab [14].

For Na^+^, there was a statistically unacceptable correlation between the two instruments. Examination of the data revealed that in 29 of the 60 (48%) cases, the Falcor generated Na^+^ values lower than those of the KoneLab. The methodology employed by the Falcor (indirect ISE) has been reported to generate lower results than the direct ISE methodology employed on the KoneLab [15, 16]. This cannot, however, explain the unacceptable correlation observed, since the results for K^+^ which were also obtained by application of the different methodologies (indirect versus direct ISE) showed an excellent correlation (*r* = 0.97). The reason for the unacceptable correlation observed for Na^+^ is not readily apparent and may merit further investigation. Similar differences in the correlations between the Falcor and KoneLab instruments for Na^+^ and K^+^ values have also been reported in a recent study which utilised samples from 60 canine (Na^+^; *r* = 0.41, K^+^; *r* = 0.96) and 60 feline (Na^+^; *r* = 0.61, K^+^; *r* = 0.97) clinical cases [14].

For urea, there was a statistically unacceptable correlation between the two instruments. The regression plot revealed 3 data points which deviated markedly from the calculated line of best fit as the Falcor values were unexpectedly higher than those obtained from the KoneLab (41.5 versus 3.1, 14.6 versus 5.2, and 13.3 versus 4 mmol/L) (Figure 1(c)). Examination of the difference plot identified only one outlier (41.5 versus 3.1 mmol/L) with the Falcor urea concentration being higher than that obtained from the KoneLab. When urea was remeasured in 2 of the 3 samples, the Falcor generated values which were similar to those obtained originally by the KoneLab (4.8 versus 3.1, 5.7 versus 5.2 mmol/L). The reason for this discrepancy in measurements of urea by the Falcor was not apparent as retrospective investigations on sample quality, operator, or instrument errors revealed no abnormalities. Reassessment of the correlation between the two instruments after exclusion of the 3-deviated points produced an acceptable correlation (*r*_*s*_ = 0.81) while difference plots analysis revealed good agreement with no outliers. Even so, this inconsistency merits further investigation as similar findings have been reported in a very low number of samples from canine (1 of 60) and feline (3 of 60) cases [14].

For ALP, there was an excellent correlation between the two analysers, but the slope value in the regression equation revealed that the Falcor ALP values were two and a half times higher. This observation was confirmed by the difference plot analysis which revealed a poor agreement with a true large positive bias of 237 iu/L. Examination of the data revealed that the 4 samples with KoneLab ALP values (526, 527, 488, and 425 iu/L) outside the reference interval established by the KoneLab (120–400 iu/L) also generated increased Falcor ALP values (1169, 1121, 1398, and 1223 iu/L). The higher ALP values are most likely due to the different buffers (diethanolamine (DEA) versus 2-amino2-1-propanol buffer (AMP)) used in the methodologies of the two analysers (Table 1). DEA buffer can generate higher rates of activity than the AMP buffer [17]. Similar differences in ALP values have also been reported in studies which employed the two methodologies to analyse samples from canine and feline clinical cases [6, 14].

## 5. Conclusions

The authors believe that the Falcor is easy to handle and that a familiarisation period of one week is sufficient for routine use in general practice. For 8 of the 12 analytes (albumin, AST, CK, phosphate, K^+^, TB, creatinine, and TP), the two instruments produce values that are closely related to each other (good correlation) and are sufficiently similar to allow them to be used interchangeably without the need for additional correction factor computations (good agreement). Good correlation and agreement are also demonstrated for urea values although the Falcor in 3 samples generated inconsistent results. Because of differences in the methodologies, the Falcor results for ALP, tCa, and Na^+^ cannot be used interchangeably with those generated by the KoneLab and should be interpreted using reference intervals established from the Falcor analyser.

## Figures and Tables

**Table 1:** Methodologies used by the Falcor and KoneLab analysers.

Analyte	Falcor	KoneLab
Urea	Urease method (A. Menarini Diagnostics)	Urease method (Thermo Electron Co.)
Creatinine	Jaffé method (A. Menarini Diagnostics)	Jaffé method (Thermo Electron Co.)
TP	Biuret method (A. Menarini Diagnostics)	Biuret method (Biostat Diagnostic Systems)
Albumin	Bromocresol Green (BCG) (A. Menarini Diagnostics)	Bromocresol Green (BCG) method (Thermo Electron Co.)
CK	IFCC-optimised method at 37°C (A. Menarini Diagnostics)	IFCC-optimised method at 37°C (Thermo Electron Co.)
AST	IFCC method at 37°C (A. Menarini Diagnostics)	IFCC method at 37°C (Thermo Electron Co.)
ALP	DGKC method at 37°C, diethanolamine (DEA) (A. Menarini Diagnostics)	IFCC method at 37°C, 2-amino-2-1-propanol (AMP) buffer (Thermo Electron Co.)
TB	Acid diazo coupling method (A. Menarini Diagnostics)	Acid diazo coupling method (Thermo Electron Co.)
tCa	Cresolphthalein complexone method (A. Menarini Diagnostics)	Arsenazo III method (Thermo Electron Co.)
Phosphate	Ammonium molybdate method (A. Menarini Diagnostics)	Ammonium molybdate method (Thermo Electron Co.)
Na^+^	Indirect ISE (A. Menarini Diagnostics)	Direct ISE (Thermo Electron Co.)
K^+^	Indirect ISE (A. Menarini Diagnostics)	Direct ISE (Therno Electron Co.)

TP: total proteins, CK: creatine kinase, ALP: alkaline phosphatase, AST: aspartate aminotransferase, TB: total bilirubin, tCa: total calcium, Na^+^: sodium, K^+^: potassium.

**Table 2:** Median (range) values and Deming's regression analysis results for all analytes measured in 60 equine blood samples.

Analyte	Falcor median	Range	Konelab median	Range	Slope estimate	95% CI	Intercept estimate	95% CI	*r*_*s*_
Albumin	36	15 to 41	33	15 to 39	1.13	1.03 to 1.23	−2.24	−5.53 to 1.06	0.93
ALP	350	155 to 1398	161	88 to 527	2.64	2.45 to 2.84	−79.6	−121.7 to −37.4	0.94
AST	315	194 to 7353	351	207 to 8183	0.90	0.90 to 0.91	9.91	3.71 to 16.1	0.98
tCa	3.18	2.56 to 5.62	3.04	2.40 to 4.58	1.53	1.30 to 1.76	−1.46	−2.16 to −0.76	0.71
CK	247	73 to 3959	265	77 to 4435	0.89	0.88 to 0.90	16.2	8.29 to 24.1	0.98
Creatinine	117	60 to 167	119	65 to 194	1.02	0.86 to 1.18	−8.75	−28.5 to 11.0	0.88
Phosphate	1.02	0.40 to 2.38	0.95	0.29 to 2.23	0.99	0.93 to 1.05	0.10	0.03 to 0.16	0.95
K^+^	3.45	2.44 to 5.79	3.81	2.92 to 7.06	0.89	0.83 to 0.94	0.05	−0.17 to 0.28	0.97
Na^+^	137	129 to 148	138	128 to 144	1.50	1.08 to 1.92	−69.0	−126.7 to −11.4	0.68
TB	29	12 to 96	33	14 to 100	0.93	0.89 to 0.98	0.58	−1.47 to 2.63	0.98
TP	65	47 to 91	64	46 to 90	1.03	0.93 to 1.13	−0.38	−6.83 to 6.07	0.92
Urea	6.2	3.2 to 41.5	5.1	2 to 8.9	−148	−1795 to 1498	5.22	−8003 to 9548	0.64

CI: confidence intervals, TP: total proteins, CK: creatine kinase, ALP: alkaline phosphatase, AST: aspartate aminotransferase, TB: total bilirubin, tCa: total calcium, Na^+^: sodium, K^+^: potassium.

**Table 3:** Data from Bland-Altman difference plots used to determine agreement between the Falcor and KoneLab analysers.

Analyte	Bias	95% CI for the bias
Albumin	1.9	1.5 to 2.3
ALP	237	196 to 278
AST	−54	−84 to −24
tCa	0.2	0.1 to 0.2
CK	−35	−58 to −12
Creatinine	−6	−9.2 to −2.9
Phosphate	0	0 to 0.1
K^+^	−0.4	−0.4 to −0.3
Na^+^	−0.2	−0.8 to 0.5
TB	−2.2	−3.2 to −1.1
TP	1.4	0.7 to 2.2
Urea	1.8	0.5 to 3.1

CI: confidence intervals, TP: total proteins, CK: creatine kinase, ALP: alkaline phosphatase, AST: aspartate aminotransferase, TB: total bilirubin, tCa: total calcium, Na^+^: sodium, K^+^: potassium.
